# Supplementary material for: Prevalence and factors associated with anogenital warts among sexual and gender minorities attending a trusted community health center in Lagos, Nigeria
Source: PLOS Glob Public Health. 2022 Nov 8;2(11):e0001215. doi: 10.1371/journal.pgph.0001215 (PMC10021808; doi:10.1371/journal.pgph.0001215)
Supplement: S1 Table — (DOCX) [file pgph.0001215.s001.docx]

| **S1 Table: Sensitivity comparison of sociodemographic and sexual behaviour factors between SGM included and excluded from the analyses** | | | |
| --- | --- | --- | --- |
| **Characteristics (N=672)** | **Included participants (n=478)** | **Excluded participants (n=194)** | **p-value** |
| **Enrolment period** | 13may2014 – 29sep2016 | 07may2014 – 28sep2016 |  |
| **Age in years – median (IQR)** | 22 (20 - 26) | 22 (20 – 25) | 0.08 ^K^ |
| **Age of sexual debut – mean (SD)** | 16.8 (3.6) | 17.1 (3.5) | 0.61 ^B^ |
| **Marital status – n (%)** |  |  | 0.31 ^C^ |
| Single | 434 (90.8) | 183 (94.3) |  |
| Married/cohabiting | 30 (6.3) | 8 (4.1) |  |
| Separated/divorced/widowed | 14 (2.9) | 3 (1.6) |  |
| **Education level – n (%)** |  |  | 0.40 ^C^ |
| None/primary | 3 (0.6) | 0 (0.0) |  |
| Secondary | 337 (70.5) | 143 (73.7) |  |
| Tertiary | 138 (28.9) | 51 (26.3) |  |
| **Occupational status – n (%)** |  |  | 0.76 ^C^ |
| Unemployed | 205 (42.9) | 88 (45.4) |  |
| Student | 135 (28.2) | 55 (28.3) |  |
| Employed | 138 (28.9) | 51 (26.3) |  |
| **Gender – n (%)** |  |  | 0.41 ^C^ |
| Cisgender man | 374 (78.2) | 148 (76.3) |  |
| Transgender woman | 71 (14.9) | 26 (13.4) |  |
| Non-binary | 33 (6.9) | 20 (10.3) |  |
| **Sexual position with male partners** |  |  | 0.02 ^C^ |
| Engaged in insertive sex only | 75 (15.7) | 48 (24.7) | 0.11 ^Z^ |
| Engaged in insertive and receptive sex | 268 (56.1) | 93 (48.0) | 0.09 ^Z^ |
| Engaged in receptive sex only | 135 (28.2) | 53 (27.3) | 0.45 ^Z^ |
| **Condom use with male sexual partners in past 12 months** |  |  | 0.03 ^C^ |
| Always use condoms | 221 (46.2) | 97 (50.0) | 0.27 ^Z^ |
| Condomless during receptive sex only | 110 (23.0) | 35 (18.0) | <0.54 ^Z^ |
| Condomless during insertive sex only | 37 (7.8) | 27 (14.0) | <0.01 ^Z^ |
| Condomless during both insertive and receptive sex | 110 (23.0) | 35 (18.0) | <0.54 ^Z^ |
| **Multiple male sexual partners in past 12 months** |  |  | 0.07 ^C^ |
| No | 30 (6.3) | 20 (10.3) |  |
| Yes | 448 (93.7) | 174 (89.7) |  |
| **Transactional sex in past 12 months** |  |  | 0.25 ^C^ |
| None | 199 (41.6) | 74 (38.1) |  |
| Received money/gifts only | 163 (34.1) | 73 (37.6) |  |
| Paid money/gifts only | 47 (9.8) | 12 (6.2) |  |
| Both paid & received money/gifts | 69 (14.5) | 35 (18.1) |  |
| **Lubricant use during sex** |  |  | 0.92 ^C^ |
| No | 31 (6.5) | 13 (6.7) |  |
| Yes | 447 (93.5) | 181 (93.3) |  |
| **HIV status** |  |  | <0.01 ^C^ |
| Without HIV | 136 (28.5) | 84 (43.3) | <0.01 ^Z^ |
| Living with HIV | 342 (71.5) | 62 (32.0) | <0.01 ^Z^ |
| Missing data | 0 (0.0) | 48 (24.7) | <0.01 ^Z^ |

^K^ Kruskal-Wallis test for medians; ^B^ Bartlett's test of homogeneity of variances; ^C^ Chi-square test for homogeneity; ^Z^ Two-sample Z-test of proportions; ^IQR^ Interquartile range; ^SD^ Standard deviation; ^n (%)^ Proportion and column percentage.
