# Supplementary material for: Prevalence and factors associated with anogenital warts among sexual and gender minorities attending a trusted community health center in Lagos, Nigeria
Source: PLOS Glob Public Health. 2022 Nov 8;2(11):e0001215. doi: 10.1371/journal.pgph.0001215 (PMC10021808; doi:10.1371/journal.pgph.0001215)
Supplement: S2 Table — (DOCX) [file pgph.0001215.s002.docx]

| **S2 Table: Bivariate analysis of condom-use and HIV status among SGM** | | | | |
| --- | --- | --- | --- | --- |
| **Characteristics N=478** | **n[%]** | **PLWH**  **n(%)** | **PWTH**  **n(%)** | **p-value** |
| **Condomless sex with male sexual partners in past 12 month** |  |  |  |  |
| Always use condoms | 221 [44.0] | 145 (59.4) | 76 (40.6) | 0.060 |
| Condomless during receptive sex only | 110 [23.8] | 26 (51.8) | 11 (48.2) |  |
| Condomless during insertive sex only | 37 [11.1] | 92 (84.5) | 18 (15.5) |  |
| Condomless during both insertive and receptive sex | 110 [21.1] | 79 (65.6) | 31 (34.4) |  |
| PLWH – People living with HIV; PWTH – People without HIV; n[%] – frequency [column percentage]; n(%) – frequency (row percentage); statistical significance is p-value <0.05 | | | | |
