# Supplementary material for: Prevalence and factors associated with anogenital warts among sexual and gender minorities attending a trusted community health center in Lagos, Nigeria
Source: PLOS Glob Public Health. 2022 Nov 8;2(11):e0001215. doi: 10.1371/journal.pgph.0001215 (PMC10021808; doi:10.1371/journal.pgph.0001215)
Supplement: S3 Table — (DOCX) [file pgph.0001215.s003.docx]

| **S3 Table: Binary logistic regression of selected factors and anogenital warts among SGM living with HIV** | | | |
| --- | --- | --- | --- |
| **Factors (n = 342)** | **n** | **Crude OR (95%CI)** | **p value** |
| **Condomless sex with male sexual partners in past 12 month** |  |  |  |
| Always use condoms | 145 | ref. |  |
| Condomless during receptive sex only | 92 | 0.79 (0.24 – 2.57) | 0.698 |
| Condomless during insertive sex only | 26 | 0.52 (0.12 – 2.22) | 0.382 |
| Condomless during both insertive and receptive sex | 79 | 1.52 (0.59 – 3.85) | 0.377 |
|  |  |  |  |
| **ART** |  |  |  |
| No | 253 | ref. |  |
| Yes | 89 | 1.97 (0.75 – 5.19) | 0.166 |
|  |  |  |  |
| **Viral load** |  |  |  |
| ≤1000 copies/mL | 123 | ref. |  |
| >1000 copies/mL | 219 | 0.84 (0.36 – 1.93) | 0.691 |

OR – Odds ratio ref. – referent group; CI – confidence interval; Statistical significance is p-value <0.05
