# Supplementary material for: Prevalence and factors associated with anogenital warts among sexual and gender minorities attending a trusted community health center in Lagos, Nigeria
Source: PLOS Glob Public Health. 2022 Nov 8;2(11):e0001215. doi: 10.1371/journal.pgph.0001215 (PMC10021808; doi:10.1371/journal.pgph.0001215)
Supplement: Attachment — Submitted filename: Response to Reviewers Letter_PLOS_11.09.2022.docx [file pgph.0001215.s004.docx]

August 21, 2022

Abram L. Wagner, PhD, MPH

Academic Editor

PLOS Global Public Health

Dear Dr. Wagner,

**PGPH-D-22-00848 – Response-to-reviewers’ Letter**

**Prevalence and factors associated with anogenital warts among sexual and gender minorities attending a trusted community health Center in Lagos, Nigeria**

Thank you very much for sharing the concerns of the reviewers of our manuscript titled: ‘Prevalence and factors associated with anogenital warts among sexual and gender minorities attending a trusted community health Center in Lagos, Nigeria’.

These have been carefully reviewed, and all the concerns have been addressed as follows:

**Journal Requirements:**

1. (i) Please amend your detailed online Financial Disclosure statement. This is published with the article. It must therefore be completed in full sentences and contain the exact wording you wish to be published.

**Response**: This is noted with thanks. The online Financial Disclosure statement has been amended.

(ii) State what role the funders took in the study. If the funders had no role in your study, please state: “The funders had no role in study design, data collection and analysis, decision to publish, or preparation of the manuscript.”

**Response**: The U.S. Army was one of several entities that provided funding for this research. U.S. Army investigators were involved in the study design, study operations, data collection, data analysis, data interpretation, and writing of the report. This will be stated online.

2. Please update your online Competing Interests statement. If you have no competing interests to declare, please state: “The authors have declared that no competing interests exist.”

**Response**: The online Competing Interests statement will be updated to indicate that we have no competing interests to declare.

3. We do not publish any copyright or trademark symbols that usually accompany proprietary names, e.g. (R), (C), or TM (e.g. next to drug or reagent names). Please remove all instances of trademark/copyright symbols throughout the text, including ® (Determine®, Uni-gold®) on page 5.

**Response**: Thanks for the information. All trademarks and copyright symbols have been removed.

4. Please review your reference list to ensure that it is complete and correct. If you have cited papers that have been retracted, please include the rationale for doing so in the manuscript text, or remove these references and replace them with relevant current references. Any changes to the reference list should be mentioned in the rebuttal letter that accompanies your revised manuscript. If you need to cite a retracted article, indicate the article’s retracted status in the References list and also include a citation and full reference for the retraction notice.

**Response**: This is noted with thanks. The reference list has been cross-checked for correctness and completeness. Some of the references were deleted from the manuscript while others were added.

The deleted references include:

- Blas MM, Brown B, Menacho L, Alva IE, Silva-Santisteban A, Carcamo C. HPV Prevalence in Multiple Anatomical Sites among Men Who Have Sex with Men in Peru. PLoS One. 2015;10(10):e0139524.
- {Heinze, 2018 #242}
- Galea JT, Kinsler JJ, Galan DB, Calvo G, Sánchez H, Leon SR, et al. Factors Associated With Visible Anogenital Warts Among HIV-Uninfected Peruvian Men Who Have Sex With Men and Transwomen:A Cross-Sectional Study. Sexually Transmitted Diseases. 2015;42(4):202-7.
- Koshiol JE, Laurent, S. A., Pimenta, J. M. Rate and predictors of new genital warts claims and genital warts-related healthcare utilization among privately insured patients in the United States. Sexually Transmitted Diseases. 2004;31(12):748-52.
- Fernández-Nestosa MJ, Cubilla AL. The natural history of HPV infection in men. Nature Reviews Urology. 2015;12(9):478-80.

The new references cited in the revised manuscript include:

- Baral SD, Ketende S, Schwartz S, Orazulike I, Ugoh K, Peel SA, et al. Evaluating Respondent-Driven Sampling as an Implementation Tool for Universal Coverage of Antiretroviral Studies Among Men Who Have Sex With Men Living With HIV. J Acquir Immune Defic Syndr. 2015;68(2):107-13.
- {Epstein, 2014 #248}
- {Drückler, 2022 #250}
- {Van Gerwen, 2020 #253}

**Reviewer # 1:**

(1) In discussing the higher prevalence of anogenital warts among men living with HIV, the authors remark that “HIV may further exacerbate the presentation of [216] anogenital warts, as it has been associated with reactivation and lower immune control of latent [217] HPV infection.(28),” but was there more (condomless) receptive anal sex among HIV-positive study participants that may also explain the relationship with rates of anogenital warts?

**Response**: We performed exploratory bivariate analyses of condom use among SGM living with HIV (supplementary table 3), and found there was no significant distribution in their weighted proportions. Hence, condomless receptive anal sex may not explain the association between HIV and anogenital warts in our study. We have added a sentence to explain this in the manuscript. See lines 177-180:

*Further exploratory bivariable analyses of our data did not show associations between anogenital warts and HIV (supplementary table 2) other covariates including condom use, ART use, and viral suppression (Supplementary Table 3).*

(2) There is passing reference to the existence of HPV vaccines. What access (or barriers to access) are there to vaccination in the study area?

**Response**: Although HPV vaccines are licensed for use in Nigeria, unfortunately there are currently no organized Government endorsed immunization programs targeted at boys. We believe our study will contribute to strengthening the evidence-base to influence national HPV prevention and treatment programs in Nigeria. This has been added to the manuscript on lines 287-288: *Furthermore, targeted HPV vaccination of SGM boys before their sexual debut would be a cost-effective strategy with significant impact.*

**Reviewer 2:**

**Methods/Statistical analysis:**

•The data was collected using RDS, but all percentages are presented without adjusting for bias in sample collection method. Please explain and include information on potential bias due to the sampling method. Also, mention if the differences found between adjusted and unadjusted prevalence were large.

**Response**: Thank you for this very important observation. We have reanalyzed our data using RDS weighting based on the network size and number of recruitment seeds. We also included the recruitment waves, which showed high depths (up to 26) recommended to reduce bias. Furthermore, we explored if this could confound the prevalence estimates. We have reported the adjusted prevalence of anogenital warts in the manuscript as 54% instead of 53% (a difference of 1%). We have also applied the RDS weighting in our bivariate and multivariable analyses and have reported the revised results.

Lines 129-132: *RDS weighting was used in our analyses based on individual social network size and the number of coupons distributed. Weighted Pearson’s chi-squared test was used to compare the differences in the prevalence of anogenital warts across various demographic, biological, and behavioral characteristics.*

• The definition of the variable “occupational status” is not clear. How students who worked were classified?

**Response**: We have taken out students from categorization of occupational status and created a variable “currently a student” [Yes or No], which we included in our analyses. Occupational status is now classified as employed vs. unemployed.

Lines 124-125: *viral load, CD4 count, age, age at sexual debut, marital status, education, occupation, currently a student,*

• Some independent variables are likely correlated with one another such as age and being a student; and ART, viral load and CD4 count. It is not clear how confounders were managed in the multivariate analysis.

**Response**: After reanalyzing our data using the RDS sampling weights, potential collinear variables that were initially included in our multivariable model, such as age and being a student, where no longer included because they did not meet the variable-selection criterion of p<0.157 in the bivariate analysis. As such, we do not have any collinearity issues in our final multivariable analysis. Furthermore, ART, viral load, and CD4 count, were not considered for inclusion into our multivariable model because our sample population also included people without HIV.

• The AOR for those on ART vs. not on ART were not presented. Any reason for that?

**Response**: We did not include ART in our multivariable model, because our study population included people without HIV, in whom ART status was not a consideration. However, we conducted a bivariable analysis for people living with HIV, which included ART status (supplementary table 3) and found no statistical significance.

**Discussion**

• The lack of association between anogenital warts and viral load in the study warrant some explanation, as it does not seem to be related to the sample size as for CD4 count. Additionally, suggest adding an explanation for the counterintuitive result of those on ART to have higher prevalence of warts.

**Response**: We have added some sentences in the paragraph to explain the lack of association between anogenital warts and viral load and an explanation has been provided for the higher prevalence of warts among SGM on ART. A supplementary table 3 has also been included to show no associations between ART, viral load and anogenital warts.

Lines 252-262: *Our study may have been underpowered to evaluate HIV-related immunosuppression and prevalence of anogenital warts, as only 9% of the sample reported a CD4 count of <200 cell/mm3. Using a single CD4 measurement that is prone to internal and external influences, may result in misclassification of immune status. CD4 trajectories from longitudinal data are recommended as better predictors of incident anogenital warts among persons living with HIV.(29) Lastly, the lack of association between anogenital warts and viral load could be due to the stage of infection with many participants being newly diagnosed and are yet to experience chronic HIV.*

• Several results can lead to recommendations for programme managers, as the excellent one on wart management for PLHIV mentioned in the conclusion. Regarding other recommendations made, specific suggestions are:

a. Behavioral risk interventions: It is not clear what is being recommended regarding behavioral risk interventions that could address anogenital warts beyond what is already addressed (i.e, condom and lubricant use). Suggest deleting this recommendation by deleting the sentence on lines 207-208.

**Response**: Thank you for this suggestion. We have deleted the sentence on lines 207-208.

b. Condom promotion and access. Good point. On line 235 add “and” (…use of condom and compatible water-based lubricants). Add in the conclusion.

**Response**: This was an omission. ‘And’ has been added to complete the sentence ‘…use of condom and compatible water-based lubricants’…A sentence has also been included in the conclusion.

Line 271-273: *Health interventions for SGM in Nigeria that increase access to and promote the use of condom and compatible water-based lubricants may maintain the integrity of the mucosal epithelial barrier.*

Line 286-287: *Health interventions for SGM in Nigeria should include access to condoms and compatible water-based lubricants.*

c. Vaccination of boys. I am not convinced that this would be the most effective strategy as vaccination coverage at populational level would have to be very high to reach SMG before sexual debut. Suggest focusing on catch-up targeted vaccination for SMG in friendly clinics instead. The rational is the likelihood of co-infection with multiple viral HPV genotypes and the potential cross-protection effect when quadrivalent or nonavalent vaccines are used (see US CDC recommendations for vaccinating all adults).

**Response**: The suggestion is well received. However, although vaccination of boys may not be the most cost-effective strategy, we agree that targeted vaccination of SGM may be a higher priority approach. The manuscript has been modified accordingly.

Line 287-288: *Furthermore, targeted HPV vaccination of SGM boys before their sexual debut would be a cost-effective strategy with significant impact.*

d. Long-term sequelae (line 248-249) goes beyond the scope of the paper. The sentence could be deleted and replaced by a adding a sentence the need to address anogenital warts to improve quality of life, including sexual life.

**Response**: The manuscript has been revised based on the guidance provided to include quality of health.

Line: 283-286: *Offering warts management services as an inclusive component of comprehensive HIV prevention, treatment, and care services may alleviate the high physical, psychosocial and economic burden of anogenital warts. This will go a long way in improving the quality of life of SGM.*

**Minor points:**

• In the introduction, besides mentioning the STI-related stigma and discrimination, suggest mentioning the “impact” of anogenital on quality of life. This is best described among women, but some small studies have been addressing this important health consequence.

**Response**: Impact on quality of life has been incorporated in the manuscript. Line 283-286: *Offering warts management services as an inclusive component of comprehensive HIV prevention, treatment, and care services may alleviate the high physical, psychosocial and economic burden of anogenital warts. This will go a long way in improving the quality of life of SGM.*

• The list of independent variables is not complete. Suggest either explaining why some were not mentioned or refer to the complete list on table.

**Response**: This was an omission. All the independent variables have been added. Lines 114-146: *Sexual orientation was categorized as homosexual, bisexual, heterosexual, or queer; gender identity was categorized as cisgender, transgender and non-binary based on participants’ self-reported responses. Sexual position with male partners was categorized as: ‘insertive sex only’, ‘receptive sex only’, or ‘both insertive and receptive sex’. Sexual positions with female partners were assessed and categorized separately. Condom use was defined as always used condoms or any condomless sex during insertive and/or receptive sex with both male and female partners in the preceding 12 months. In these analyses, multiple sexual partners were defined as having more than one male or female partner in the preceding 12 months. Transactional sex identified participants who did or did not exchange money and/or gifts for sex in the preceding 12 months. The use of and ease of access to lubricants during sex were also assessed, as well as HIV, ART use, viral load, CD4 count, age, age at sexual debut, marital status, education, occupation, currently a student, and study recruitment waves categorized by fives (0-4,5-9,10-14,15-19,20-24,25-29).*

• Line 154, rephrase “The 478 participants who were included in these analyses had similar characteristics to the 194 155 participants who were excluded…” to “The 478 participants who were included in these analyses had similar characteristics to the 194 155 participants who were excluded, with the exception of condomless insertive sex only and…..

**Response**: Thanks for this suggestion. The sentence has been rephrased. Lines 162-167: *The 478 participants who were included in these analyses had similar characteristics to the 194 participants who were excluded (except for condomless insertive only and HIV status) due to incomplete behavioral data or non-engagement with the warts management service (Supplementary Table 1). However, the excluded participants were more likely to report condomless insertive sex only (7.8% vs. 14.0%, p=0.01), be living without HIV (43.3% vs. 28.5%, p<0.01), or have unknown HIV status (24.7% vs. 0, p<0.01).*

• In the conclusion, suggest changing “The prevalence of anal warts among SGM in Nigeria is high..” to “seems high”, considering the inherited bias of the study addressed in the limitations. Lines 282-283: *The prevalence of anal warts among SGM in Nigeria seems high, particularly among those living with HIV.*

**Response**: The sentence has been modified as suggested. Thank you very much.
